# Enabling middle‐aged and older adults accessing community services to reduce social isolation: Community Connectors

**DOI:** 10.1111/hsc.13228

**Published:** 2020-12-08

**Authors:** Clarissa Giebel, Shaima Hassan, Gina Harvey, Conal Devitt, Lesley Harper, Cheryl Simmill‐Binning

**Affiliations:** ^1^ Department of Primary Care & Mental Health University of Liverpool Liverpool UK; ^2^ NIHR ARC NWC Liverpool UK; ^3^ Community Connectors Sefton Council for Voluntary Service Liverpool UK; ^4^ Health Innovation Campus Team Lancaster University Lancaster UK

**Keywords:** health inequalities, loneliness, older adults, public involvement, social connectedness, social isolation

## Abstract

A large number of older adults (65+ years) live on their own, and can experience high levels of loneliness. However, accessing activities to engage with their community can be difficult either due to their age and associated comorbidities, such as frailty, or due to financial reasons, for lacking the funds to access transport to activities. The aim of this study was to evaluate an existing service in the North West of England, *Community Connectors*, which enables people aged 18 and above to access social activities within their community in order to reduce loneliness and social isolation. This study only included middle‐aged and older adults. A total of 13 semi‐structured interviews were performed after people had taken part in the 14‐week Community Connectors programme. Data were coded by two research team members by using thematic analysis. Members of the public were involved in the design of this study, and in the dissemination. Between June 2017 to September 2018, 234 older adults and 53 middle‐aged adults were referred to Community Connectors. Four themes emerged from the interviews: falling out of society; easy self‐referral; structured supportive services; and reconnecting with community. Services often depend on individuals making the first step to access, however, without easy or facilitated access people can becoming isolated. Participants reported on how Community Connectors provided easy and open access that enabled better response to individual needs. The structured support provided individuals with confidence in engaging with community activities and enhanced individuals’ social networks. Community Connectors enables middle‐aged and older adults to engage with social activities in their community, and thus helps participants to feel less lonely and more socially connected. Future work needs to quantitatively measure the impacts of the service on loneliness, depression, and social connectedness in order to fully understand their impact.

**What is known about this topic**

- Many older adults are socially isolated
- Limited evidence on social prescribing effects
- Social engagement can reduce loneliness

**What this paper adds**

- First evidence on a social community service reducing social isolation in middle‐aged/older adults
- Community Connectors built confidence in service users
- Need for more easily‐accessible social community services

## 1 INTRODUCTION

With an increasing ageing population, the demand for health and social care services for age‐related conditions is growing. In the UK, the proportion of people aged 65 or above is steadily increasing, with currently 18.2% of the population aged 65 or older. This amounts to an older adult dependency ratio (the number of older adults per 1,000 adults of working age) of 289. Of those older adults, 3.8 million (approximately 32%) are living on their own, most of which are female (66.5%) (ONS, 2018). Therefore, it might not be surprising to find that loneliness is a common issue in older people, affecting about one third of older adults (Grenade & Boldy, 2008; Victor et al., 2005).

Loneliness has been linked to a variety of physical and mental health problems, including depression (Donovan et al., 2016), increased blood pressure (Hawkley et al., 2010), increased rates of cognitive function decline (Donovan et al., 2016), and has been linked to increased rates of mortality (Holt‐Lunstad et al., 2015; Perissonoto et al., 2012). Social isolation is often underpinning loneliness (Shankar et al., 2017), with social isolation often described as a lack of social integration (Grenade & Boldy, 2008). One tell‐tale sign of loneliness can be problems with engaging in activities of daily living, such as washing or dressing (Cohen‐Mansfield et al., 2016; Perissinotto et al., 2012; Shankar et al., 2017). Being able to initiate and perform everyday activities is important for people to stay independent in their own home. However, experiencing difficulties in engaging in daily tasks can often mean that family members will have to help their loved ones with those tasks, and in some cases paid carers will have to support the person. With functional decline being a major symptom of dementia (Giebel et al., 2018), it is important to differentiate the causes of functional decline, and to provide appropriate interventions that either target loneliness or support people with their dementia.

Interventions to reduce loneliness and social isolation in older adults have frequently been reported. In a recent scoping review, O’Rourke et al. (2018) reported on 39 interventions to reduce loneliness in older adults, categorised into nine different intervention types, including animal contact, engaging in social activities, reminiscence, and support groups. However, the review lacked evaluation of the interventions’ efficacy. By reviewing 32 interventions, Dickens et al. (2011) however showed that 86% of those interventions providing an activity and 80% of those providing some form of support resulted in positive outcomes for older adults in reducing social isolation. Overall, it is vital to engage in social activities as they are related to improved well‐being in older adults (Huxholdt et al., 2014). One important element to consider in providing social activities is to evaluate an individual's needs and wishes, and supporting them on the individual steps to engaging with an activity.

Accessing and engaging in social activities can be difficult for some older adults. For example, being frail and having physical limitations can lead to difficulties accessing suitable transport. Additionally, experiencing high levels of frailty is found to increase the risk of Alzheimer's disease pathology to develop into Alzheimer's disease (Wallace et al., 2019). Therefore, enabling older adults to engage in social activities may contribute to reducing not only their loneliness, but also their mobility problems. In addition to frailty hindering some older adults from accessing activities, the costs of getting to and attending a social activity, as well as limited public transport opportunities, might also be a barrier. These are elements that can lead to experiencing health inequalities; living in disadvantaged neighbourhoods can limit people's access and availability of recourses. Research into the relationship between socioeconomic status (SES) and social networks indicates that higher SES, particularly high levels of education, across the adult life span is associated with wider non‐family social networks in old age, whereas people from low SES have smaller networks in general (Van Groenou & van Tilburg, 2003). This is corroborated by findings from a recent review, indicating that low income and lower educational level were linked to increased levels of loneliness in older adults (Cohen‐Mansfield et al., 2016). This suggests that people from low SES may particularly require support in engaging in social community activities in order to widen their social networks.

Community Connectors (CCs) is a borough wide social support service managed by Sefton Council, located within the North West Coast, which is one of the most disadvantaged regions of England (Department for Communities & Local Transport, 2015). This third sector organisation provides social support to people aged 18 and above to access local support groups and other social activities in order to improve their well‐being and tackle loneliness by improving their social connectedness. Therefore, CCs provides a gateway to support them attending a service, which is tailored to the individual's needs. Some people may need transport from their own home, whereas others may be able to go to the local bus stop and meet one of the volunteer community champions there. Thus, CCs fills in an important role of helping people from any socio‐economic background to access social activities.

The aim of this study was to evaluate whether accessing CCs reduces loneliness and social isolation in older adults. This study forms part of the National Institute for Health Research (NIHR) Collaboration for Leadership in Applied Health Research and Care North West Coast (CLAHRC NWC) Partner Priority Programme (PPP). The PPP gathers together academics, partners from NHS organisations, local authorities and clinical commissioning groups (CCGs), as well as members of the public, to jointly co‐produce and co‐design evaluations of ongoing services, with the aim of implementing effective services subsequently on a wider scale across the North West Coast. While other studies have looked into interventions aimed at reducing loneliness and social isolation in older adults (Banks & Banks, 2002; Pitkala et al., 2009; Winstead et al., 2014), Community Connectors exists in the community and slightly varies from tested interventions. The service provides a bridge between older adults and social activities, by actively supporting them to become more independent and access social activities in their own community, and supporting them to age well. Therefore, CCs addresses a key priority of the recently released NHS Long Term Plan (2019), via means of social prescribing.

### 1.1 The Community Connectors service

Community Connectors is a structured support service that enables access to local support through a range of early intervention and prevention services that already exist, many in the voluntary, community and faith (VCF) sector such as luncheon clubs, debt awareness, social activities, befriending, foodbanks, as well as commissioned services. People who are at risk of feeling lonely and isolated, with low level mental health needs, and do not meet the eligibility criteria of Adult Social Care can access the service via two ways: 1) referrals from services such as Adult Social Care, GP or Health Professional, local group or organisation, including the Stroke Association, Macmillan Information Centre, and housing associations. Individuals on the Adult Social Care caller log who would not be offered social care assessments are referred to Community Connectors for assessment on a weekly basis. 2) Self‐referrals, Community Connectors has flyers and posters visible in Borough Council Contact Centre, GP surgeries, local supermarkets, places of worship, leisure centres, libraries and on social media. To facilitate further access, Community Connectors currently has 90 registered champions, 50 of which are either active or new and awaiting training. Champions are volunteers who raise awareness of the services within the local community. Community Connectors is also represented in a local council shop and provides a drop‐in service for easy access and self‐referral for individuals seeking support and signposting.

Once the individual is referred whether they are self‐referred or referred from Adult Social Care or GPs to community connectors, their needs are assessed within 48 hr of referral, and subsequently paired with a community champion. The community champion goes out into the community and provides person‐centred support, depending on their individual needs and activity preferences. On a lower level, volunteers meet and greet the person at a local organisation. On a medium level, volunteers might pick up basic shopping. On a high input level, volunteers provide home help, accompany people on public transport, and accompany people to shops.

This service is a 14‐week programme, during which the Champion meets with the client regularly, and the CC meets with the client around Week 7 and Week 14 to assess the progress of the individual.

## 2 METHODS

### 2.1 Design

This service evaluation employed a qualitative study design using semi‐structured one‐to‐one interviews to explore services users’ experiences and impact of CCs.

### 2.2 Participants and recruitment

The study used purposeful sampling to recruit service users who completed the 14‐week programme with CCs. At total of 13 participants were recruited by having approached individuals aged 50+. All participants that were approached agree to take part. At the beginning of their interview, participants provided written informed consent.

### 2.3 Data collection

An interview guide was co‐designed via public involvement. The topic guide included a set of open questions that explored participants’ reasons for seeking support, how they accessed servicers, barriers to access or support, what benefits taking part in the programme had and what changed following their involvement with CCs. The duration of each interview ranged from 30 to 40 min and all interviews were audio recorded. Data were collected until no new, or repetitive, information emerged from the interviews, and data collection took place in early 2018. While the programme lasted 14 weeks, participants were interviewed any time period after their 14‐week engagement. Participants were interviewed by a trained member of CCs who undertook an internship within the CLARHC NWC.

### 2.4 Data analysis

Data were transcribed and analysed using a thematic approach as a framework to handle raw data (Braun and Clark, 2006), with transcripts being checked for accuracy and omissions. Following each interview, audio recordings were transcribed for data analysis. Both an academic and a member of Community Connectors made notes of particular themes from each transcript, and using Nvivo11 data were revisited to identify patterns and potential themes. Final themes were identified and discussed with the research team including public advisers.

### 2.5 Ethical consideration

Ethical approval was obtained from both the Community Connectors commissioners and Lancaster University Faculty of Health and Medicine Research Ethics Committee (Reference number: FHMREC18045). Written and informed ‘process consent’ was considered throughout this study; verbal and written consent was obtained.

### 2.6 Public involvement

Two members of the public were recruited by Community Connectors at the beginning of the project to act as public advisers to ensure that the project was grounded in the everyday needs of those people accessing the service and providing contextual information for the interpretation of the findings. The public advisers were selected due to their considerable working knowledge of the demographics of the Sefton area, and their keen interest in loneliness and social isolation. Both public advisers were involved in the design of the project, the analysis of the findings, and in the dissemination. For this purpose, they attended regular project team meetings and CLAHRC NWC PPP workshops, to learn more about service evaluations and other research‐related skills. Public advisers received a fee according to NIHR INVOLVE [23] guidelines for each project activity they took part in, and had their travel expenses reimbursed.

## 3 RESULTS

### 3.1 Demographics

Between June 2017 and September 2018, Community Connectors dealt with 387 referrals of adults aged 18 and above. Of these, 67 were missing information on their age, so that only 322 cases were included. The large majority of people referred to the service were older adults aged 65 or above (*n* = 234, 72.7%), followed by middle‐aged adults (Age between 50 and 64) (*n* = 53, 16.5%). Thirty‐five people (10.9%) referred to the service were adults aged between 18 and 49. All people being referred to the service were White Caucasian.

### 3.2 Interview findings

A total of 13 interviews were conducted; participants were between 44 and 84 years old, four male and nine female. Four main themes emerged from the data: (a) falling out of society; (b) easy self‐referral; (c) structured supportive services; and 4) reconnecting with community.

#### 3.2.1 Falling out of society

Participants reported the process in which they became lonely or isolated from society. For the majority of participants this was due to age‐related health issues. These health issues limited their ability to carry out regular day‐to‐day activities in their own homes and/or maintain regular activities they used to be involved in, including having a formal job.

‘If my friend was here she'd tell you, I used to walk so fast, I used to walk anywhere I could do anything whereas I've just deteriorated with my back’ (P9)

Some participants reported that their excessive drinking was the main issue for them becoming isolated. Other participants reported for example being a carer restricted their social interaction, and living alone with no new activities only made them feel more isolated. Another participant mentioned that they stopped doing certain activities they used to because they believed it was not age appropriate.

‘I would hardly go out, I wouldn't really go anywhere so it was boring really… well I can't sleep much anyway so I drink quite a lot to go asleep’ (P5)

Participants recognised their loneliness, with some trying to stay active to prevent their situation getting worse. However, they were limited in what they could do. The majority of participants required physical support to help stay active. Seeking support was challenging for participants though due to lack of awareness of services or resources they could access. Participants reported that this made them feel more alone and put them in a situation of feeling lost and having nowhere to turn to.

‘I going for a walk at the moment, I have to have help and I feel that the experience does not encourage people to walk with me’ (P10)

#### 3.2.2 Easy self‐referral

The services available did not seek out people who are experiencing loneliness or are at risk of isolation. The individual had to make the first step in seeking supportive services or resources. Even though participants lacked awareness of where to seek support, they were still on the lookout for information that would help provide some guidance.

‘I was after sort of information regarding what I could do, ways to build up confidence that sort of thing, because I don't know of nowhere to go or I didn't sort of know where to go to get this sort of help until Community Connectors came along’ (P8)

Some participants accessed the Community Connectors through the local community and health services drop‐in shop, and the fact that the shop had a Community Connector representative located within a local public place made it easier for participants to seek help. Participants were able to walk into the shop without a formal appointment. Service users described how a local representative in the shop and its open access increased their confidence in the support available and gave them a sense of reassurance. They were able to return to the shop at any point for social interaction.

‘I was looking for friends, looking for a place of friendship and I found it here you know in the Shop. It's a place of friendship, it's a nice place when I come down the (shopping centre) I can always drop in here for a cup of tea’ (P13)

Apart from having the Community Connectors representative in a local community and health service shop for self‐referral, community champions were key in creating awareness and guiding participants to available support. Participants reported if champions had not been available locally, they would not have been able to find the support they needed and some may have ended up in a worse situation. Participants who self‐referred on to the services through a community champion reported more confidence from the initial interaction than those who were referred through GPs or Adult services. Participants reported they had to meet the community champion a few times before they became confident with the service.

‘We did go to the foodbank on many occasions and bumped into bumped into a chap called (name) who's a champion here as well and he gave me the phone numbers and names to contact…. I really was getting on our (name) nerves and (name) was getting on mine and it's his flat at the end of the day. So it was either you know sleep on a park bench or touch wood as I say I bumped into this (name) champion’ (P2)

#### 3.2.3 Structured supportive service

In addition to the referral to the main Community Connectors engagement, such as enabling someone's confidence in attending a support group themselves, there was also wrap‐around support. The support provided was experienced as structured to address their personal needs. During the initial assessment and throughout their involvement with Community Connectors, they were given the opportunity to express their needs.

Interviewer: we talked about what skills you had what assets you had, you thought of yourself as someone who could contribute to (name) Tool Shed

Respondent: yes because I’ve always liked to do that type of job (P3)

This included exploring options available to participants, activities of interest and exploring ways in which they can facilitate participants’ involvement. Community Connectors contacted other services to facilitate further support.

‘My sleep pattern wasn't very good until I could start getting help with you (community champion) to achieve going to appointments with the (specialist care) that could give me medication and also helped with some PIP forms’ (P11)

Participants reported that the proactive support provided by the community champions was very important to them. Community champions joined them during their activities but most importantly they became their companions during their engagement with the services.

‘I’ve actually been able to get out with your help and I feel better in general having someone that I know I can call or who will call on me’ (P7)

This helped individuals build their self‐confidence and empowered them to take action by themselves.

‘Well because I’ve been going further afield with (community champion), I feel more empowered to do better things and improve my life. I’ve got more confidence to do things’ (P5)

#### 3.2.4 Reconnecting with community

The goal of Community Connectors was to reconnect people with people. Participants reported that being engaged with the services enabled them to interact with other people and have the confidence to join others in their interactions.

‘Life changed a great deal. I go downstairs as often as I can when mobility allows, I meet with the other residents down there, and we get together and have a cup of tea or a game of bingo or what have you, because I know more people now, I know more of the other residents and it's not embarrassing now’ (P7)

Participants reported that this also helped them to talk about issues that mattered to them, such as mental health and not feeling stigmatised. This also helped them to become aware of their overall well‐being, with participants stating feeling generally more positive. This included improved diet, reduced alcohol intake, feeling less lonely and improved sleep patterns.

‘Speaking to them about my mental health and talking to my darts team, they do pick me up to go to darts on a Tuesday. So that's given me some encouragement to talk to people about mental health’ (P11)

‘Now I go to bed, have a sleep get up and I’ll go to work or I do the voluntary work in the shed… I’m eating better now than what I used to, I’m getting up earlier now because I’m so used to the early morning now’ (P3)

Participants reported being more active and more involved within the community as a result of engaging with Community Connectors. This started with participants undertaking activities that they felt confident in engaging in, such as cooking, knitting, and DIY. This helped them to keep motivated and give them something to look forward too.

‘I do a lot of knitting, I do knitting for charity, for the Dementia, I do the mittens for the Dementias. The (community champion) appreciates them, he takes them as I knit them’ (P9)

Participants felt positive about their experiences of the support provided, which in turn helped them to help others through volunteering. Although none of the participants in this study became volunteer community champions, some did become volunteers at the shop; the very community‐based support shop they first accessed for help. In doing so, participants felt their own well‐being had improved, particularly feeling positive about themselves and feeling helpful in contributing to the community.

‘I feel as I've got something to aim for these days and something I can look forward to meetings at the residents association and generally sort of people in general I can talk to better than I could before’ (P8)

## 4 DISCUSSION

This study suggests that CCs achieves its main goal of enabling people to engage in activities within their community and reduce loneliness and social isolation. Almost all participants reported feeling more connected with their community as a result of accessing CCs. Being more socially connected and building new social networks can help reduce loneliness, which in turn can help alleviate potential mental health problems, such as depression (Gonyea et al., 2018). As a part of feeling more connected with their community, participants also reported increased self‐confidence. Through CCs champions helping participants to access social activities that they previously might not have felt comfortable accessing by themselves, the service helped build up their confidence levels. As a result, previous participants felt more comfortable in continuing engagement in community activities and engaging with their newly found social networks.

Considering the make‐up of people who accessed CCs, the majority were aged 65 and above. This population demographic often experiences a number of comorbidities, ranging from mobility problems and high levels of frailty (Bandeen‐Roche et al., 2015) to mental health and cognitive problems (such as dementia (Alzheimer's Society, 2014)). Therefore, engaging in physical activities in itself can at times be a problem for this group. However, by taking part in CCs, participants reported being more active and engaging in more social and physical activities. This not only benefits the mental well‐being of a person, but also their physical well‐being. By engaging in more activities, older adults may experience improved performance with everyday activities (Tomioka et al., 2016) and reduced levels of frailty, as also reported in published trials of exercise interventions (de Labra et al., 2015). Tackling frailty is an important health problem affecting a large number of older adults. With high levels of frailty being linked to an increased risk of developing Alzheimer's disease dementia (Wallace et al., 2019), CCs may be one easily accessible community service which helps support middle‐aged and older adults in being more active, and potentially having some effect on frailty. Future research needs to explore how Community Connectors may contribute to potentially reducing frailty by providing pre and post measures, and therefore contributing to the qualitative evaluation of this service.

By engaging in more social activities, and feeling more connected with their communities, participants generally felt more positive about their lives and indirectly reported improved levels of wellbeing. Considering that some people were referred to the service via healthcare professionals, and others via adult social care services, among others, it is interesting to note the link between health and social care in CCs and the effects a social prescription of social engagement can have on a person's wellbeing. Social prescription is used more and more frequently as an important add‐on in clinical treatments, involving clinicians to prescribe engagement in social activities in the local community. A museum‐based social prescription intervention for older adults for example was shown to improve their psychological wellbeing (Thomson et al., 2018), thereby supporting the general link between engaging in social activities and wellbeing (Giebel et al., 2016). The present study shows the benefits a simple social service can have on the lives of older people, something that may not be achievable by receiving only clinical treatments. In particular, CCs contacts new referrals within a maximum of 48 hr, which is a substantially faster turn‐around than many other local authority social care services. One of the difficulties of the longevity of such services however is the temporary funded nature of most social activities within a community. Often, services are only funded for a short amount of time, so that once funding for an art class or breakfast club runs out, services such as Community Connectors are unable to direct their clients to the activities they would like to engage in the most.

There are some limitations to note of this study. Community Connectors is located in one of the most disadvantaged regions in the country. Therefore, clients may display different needs to those who would come from more advantaged backgrounds, and the CCs service concept may not work as well in more advantaged areas. Moreover CCs clients were 100% White Caucasian. Looking at the ethnic demographics across the region (Sefton Council, 2015), it becomes apparent that the region has a very low proportion of residents with ethnic minority backgrounds (2.6%) compared to the national population (14.6%). Therefore, future implementations of this service should be conducted in regions with a more representative population demographic, and actively ensuring to include people from minority ethnic backgrounds. In terms of the purposeful sampling recruitment strategy, it is also to note that there is a possibility that only those participants agreed to take part who had more positive experiences of the service. However, it is likely that people with particularly negative experiences, if indeed exist, would have felt compelled to share these also.

## 5 CONCLUSIONS

CCs appears to provide effective support to middle‐aged and older adults in accessing local social activities, and helps them to feel more socially connected, more active and less lonely. Future steps will involve a quantitative evaluation of the service, as well as producing an implementation guide so that the service can be rolled out in other regions which are more ethnically diverse. This is particularly important in the light of COVID‐19, with provision of social activities and social support services suddenly significantly reduced, which in turn affects older adults’ mental well‐being (Giebel et al., 2020). By supporting older adults to access social activities in their community, CC supports people to age better in their locality, and has positive impacts on their well‐being, thereby presenting an effective step towards addressing priorities raised in recent policy guidelines (NHS Long Term Plan, 2019).

## Data Availability

Qualitative data extracts are presented in the article to support the findings. The original transcripts are not available to the public as they may contain information that could compromise the confidentiality and anonymity of the participants.
